# Identification and characterization of Lyc e 4, a Bet v 1 homologous PR-10 protein, in tomato fruits

**DOI:** 10.1186/2045-7022-3-S3-P87

**Published:** 2013-07-25

**Authors:** A Jamin, A Wangorsch, V Mahler, AH Malczyk, D Bartel, K Foetisch, J Lidholm, S Vieths, S Scheurer

**Affiliations:** 1 Division of Allergology, Paul-Ehrlich-Institut, Langen, Germany; 2 Department of Dermatology, University Hospital of Erlangen, Erlangen-Nuremberg, Germany; 3 Thermo Fisher, Uppsala, Sweden

## Background

Allergy to tomato (*Lycopersicon esculentum*) has been reported to occur in 3-16% of food allergic subjects. Tomato allergy is frequently manifested as OAS, urticaria and gastrointestinal symptoms. So far, three tomato allergens, Lyc e 1 (profilin), Lyc e 2 (invertase) and Lyc e 3 (nsLTP) are listed in the official IUIS allergen database. Although tomato allergy is frequently associated with birch pollen allergy in Central Europe, a cross-reactive Bet v 1 homologous allergen has not yet been described in tomato fruits. The objective of the present study was the cDNA cloning, recombinant expression and immunological characterization of Bet v 1 homologous PR10-proteins from tomato fruits.

## Methods

Two Bet v 1 homologous PR10 cDNA sequences (designated Lyc e 4a and Lyc e 4b) from tomato fruit (cv *Verona*) were cloned by RT-PCR and 5'RACE. Lyc e 4 proteins were expressed in *E. coli* and purified under native conditions by Ni-NTA chromatography. Purity and secondary structure of the protein were analyzed by SDS-PAGE and CD spectroscopy. Allergen-specific IgE levels to tomato, birch pollen, rBet v 1 and rLyc e 4a were measured by ImmunoCAP^TM^ in birch pollen allergic patients with a clinical history of allergy (n=45) or tolerance (n=26) to tomato.

## Results

Lyc e 4a (Acc.No. AK224718) and Lyc e 4b (Acc.No. AK247106) showed 81% amino acid identity (aa-id) to one another and 42% and 44% aa-id to Bet v 1 (Acc.No X15877), respectively. Among PR-10 allergens from other species, the two Lyc e 4 isoforms showed highest aa-id (52% and 51%) with Pru ar 1 from apricot. Both recombinant proteins displayed secondary structures similar to those of Bet v 1. Tomato-specific IgE was detectable in 67% (30/45) of tomato/birch pollen allergic patients while 76% (34/45) showed IgE binding to Lyc e 4a and 78% (35/45) to Bet v 1. All birch pollen allergic but tomato tolerant patients displayed IgE to birch and Bet v 1, as well as to Lyc e 4a, despite their tomato tolerance. The IgE-reactivity to Lyc e 4a and Bet v 1 correlated strongly, even though we observed lower levels of IgE binding to Lyc e 4a.

## Conclusion

According to the guidelines of the IUIS allergen nomenclature subcommittee, Lyc e 4 qualifies as major allergen in tomato fruits. Serological IgE testing with Lyc e 4 showed high sensitivity but low clinical specificity in this patient population. The clinical relevance of Lyc e 4 needs to be further evaluated.

## Disclosure of interest

None declared.

